# Online Public Interest in Urological Cancers During the COVID-19 Pandemic: What Can “Dr. Google” Teach Us?

**DOI:** 10.1016/j.euros.2022.01.002

**Published:** 2022-01-17

**Authors:** Zine-Eddine Khene, Sonia Guérin, Fares Khene, Benjamin Pradère, Mathieu Roumiguié, Romain Mathieu, Géraldine Pignot, Christophe Massard, Yann Neuzillet, Guillaume Ploussard, Pierre Bigot, Alexandre De la taille, Morgan Rouprêt, Karim Bensalah, Members of the French Committee of Urologic Oncology CCAFU

**Affiliations:** a Department of Urology, Rennes University Hospital, Rennes, France; b Department of Urology, Medical University Vienna, General Hospital, Vienna, Austria; c Department of Urology, University of Toulouse, Toulouse, France; d Department of Surgical Oncology, Institut Paoli-Calmettes, Marseille, France; e Department of Medical Oncology, Centre Eugène Marquis, Rennes, France; f Department of Urology, Foch Hospital, University of Versailles-Saint-Quentin-en-Yvelines, Suresnes, France; g La Croix du Sud Hospital, Quint Fonsegrives, France; h Institut Universitaire du Cancer-Toulouse, Onocopole, Toulouse, France; i Department of Urology, University of Angers, Angers, France; j Department of Urology, Mondor Hospital, Créteil, France; k Urology, GRC 5 Predictive Onco-Uro, AP-HP, Pitie-Salpetriere Hospital, Sorbonne University, Paris, France

**Keywords:** Cancer, Urology, Mass media, Public opinion, Google Trends, Coronavirus

## Abstract

**Background:**

The coronavirus disease 2019 (COVID-19) pandemic has greatly affected health care priorities.

**Objective:**

To explore and analyse trends in public online search for urological cancers.

**Design, setting, and participants:**

We performed a retrospective analysis using the Google Health Trends online tool. Data related to urological cancer terms (“prostate cancer”, “kidney cancer”, and “bladder cancer”) were extracted. We analysed trends for the whole world and for five countries: Italy, the UK, France, Sweden, and the USA.

**Outcome measurements and statistical analysis:**

A join-point regression model was used to define significant changes in trends over time. Week percentage changes (WPCs) were estimated to summarise linear trends. The Mann-Whitney test was used to compare the search volume during the COVID-19 pandemic period (from January 2020 to April 2021) and the equivalent period of 2018 and 2019.

**Results and limitations:**

During COVID-19, worldwide online interest decreased significantly for all urological cancers, especially prostate cancer (WPC: –13.9%, *p* = 0.004; WPC: –5.4%, *p* < 0.001; and WPC: –4.3%, *p* < 0.001, for prostate, kidney, and bladder cancers, respectively). The most important decline was observed in the USA. The interest for all cancers was significantly less during the COVID-19 pandemic than in the same periods of 2018 and 2019.

**Conclusions:**

Online interest in urological cancers decreased significantly during the COVID-19 pandemic. Future studies will tell us whether this will translate into worse oncological outcomes.

**Patient summary:**

Patients are increasingly searching the Internet to get information on cancer. We explored Google queries during the COVID-19 pandemic and found that online interest decreased significantly for all urological cancers, especially prostate cancer. We do not know yet whether this will translate into worse prognosis for patients.

## Take Home Message

The coronavirus disease 2019 (COVID-19) pandemic has greatly affected health care priorities. In this study, we used a publicly available big data tool to explore the interest in search terms related to urological cancers during the COVID-19 pandemic. Our results suggest that awareness of urological cancers was significantly reduced worldwide, especially in the USA.

## 1 Introduction

The novel 2019 coronavirus (coronavirus disease 2019 [COVID-19]) was identified in December 2019 in Wuhan, China; it spread widely and affected societies and health care systems deeply. To contain the spread of COVID-19, many countries imposed restrictive measures, including suspension of nonurgent visits, and routine laboratory and imaging tests. Regarding urology, it is estimated that 40% of consultations were cancelled by patients themselves or by staff without being rescheduled [1], [2], [3].

Cancer care is an important part of daily urological practice. Several health care associations (including the European Association of Urology, the American Society of Clinical Oncology, and the American Urological Association) have made recommendations to prioritise cancer care in the context of the COVID-19 epidemic [4], [5].

About 70% of cancer patients report that the Internet is their primary source of information [6]. In this context of pandemic, our objective was to explore trends in public online search related to urological cancers. We hypothesised that the COVID-19 pandemic and lockdown measures would lead to a decrease in public interest for urological cancers.

## 2 Patients and methods

### 2.1 Data sources

Google Health Trends is a web tool owned by Google Inc. (Mountainview, CA, USA) that provides access to a representative random sample of all Google queries. The interest for a specific request is expressed by the relative search volume (RSV): the ratio between the number of queries for a specific term and the overall number of Google interrogations. The RSV ranges from 0 to 100 (100 means a very frequent Google search; a score of 0 indicates a very low interest for a subject).

The data were extracted with the pytrends open-source library that provides access to Google Trends data via an application program interface [7].

### 2.2 Data collection

We specifically queried Google Health Trends (a subdivision of Google Trends dedicated to health care) and downloaded the data related to three urological cancers (“prostate cancer”, “kidney cancer”, and “bladder cancer”). We extracted data from the whole world and those specific to five nations: Italy (the first nation where the epidemic took alarming proportions in Europe), the UK and France (two major COVID-19 outbreaks in Europe), Sweden as a nation in Europe without confinement measures, and the USA as one of the worse affected countries in the world. The data were extracted from January 12, 2020 (the day China publicly released the COVID-19 genetic sequence). We compared these data with those from the same period in 2018 and 2019.

### 2.3 Statistical analysis

Statistical analysis consisted of three steps.

First, a join-point regression (JPR) model was used to define significant changes in trends during the COVID-19 pandemic. In brief, the JPR model is a form of regression analysis in which trend data can be described by several linear segments and join points (points at which trends change). Using the log transformation, it estimates the week percentage change (WPC) and the respective 95% confidence intervals (CIs) between two join points. A positive WPC indicates an increasing trend, while a negative WPC means decreased interest. The permutation test (obtained by the grid search method) was used to determine the number of significant joint points. Data retrieved from search terms were plotted in polynomial trendlines. A full description of the JPR in the analysis of trends in cancer rates, with a specific reference to prostate cancer, has been reported by Kim and colleagues [8].

Second, the Mann-Whitney test was used to compare median RSV values during the COVID-19 pandemic period (January 12, 2020 to April 2021) with those of the corresponding time periods of 2018 and 2019.

Finally, various sensitivity analyses were performed. First, we used the module *pytrends.related_topics* to find the most relevant related searches for each urological cancers across the whole world and compared their median RSV values with those of the prior 2 yr (2018–2019). Second, an analysis testing the hypothesis that the interest of public in urological cancers was influenced by the season was conducted using an interaction term.

All statistical analyses were performed using STATA 15.1 (StataCorp, College Station, TX, USA) and Join Point Trend Analysis Software V. 4.9.0.0 (Statistical Research and Applications Branch, National Cancer Institute, Bethesda, MD, USA). A two-tailed test with *p* < 0.05 was considered statistically significant.

## 3 Results

### 3.1 Trend analysis during the COVID-19 pandemic period

**Fig. 1:**
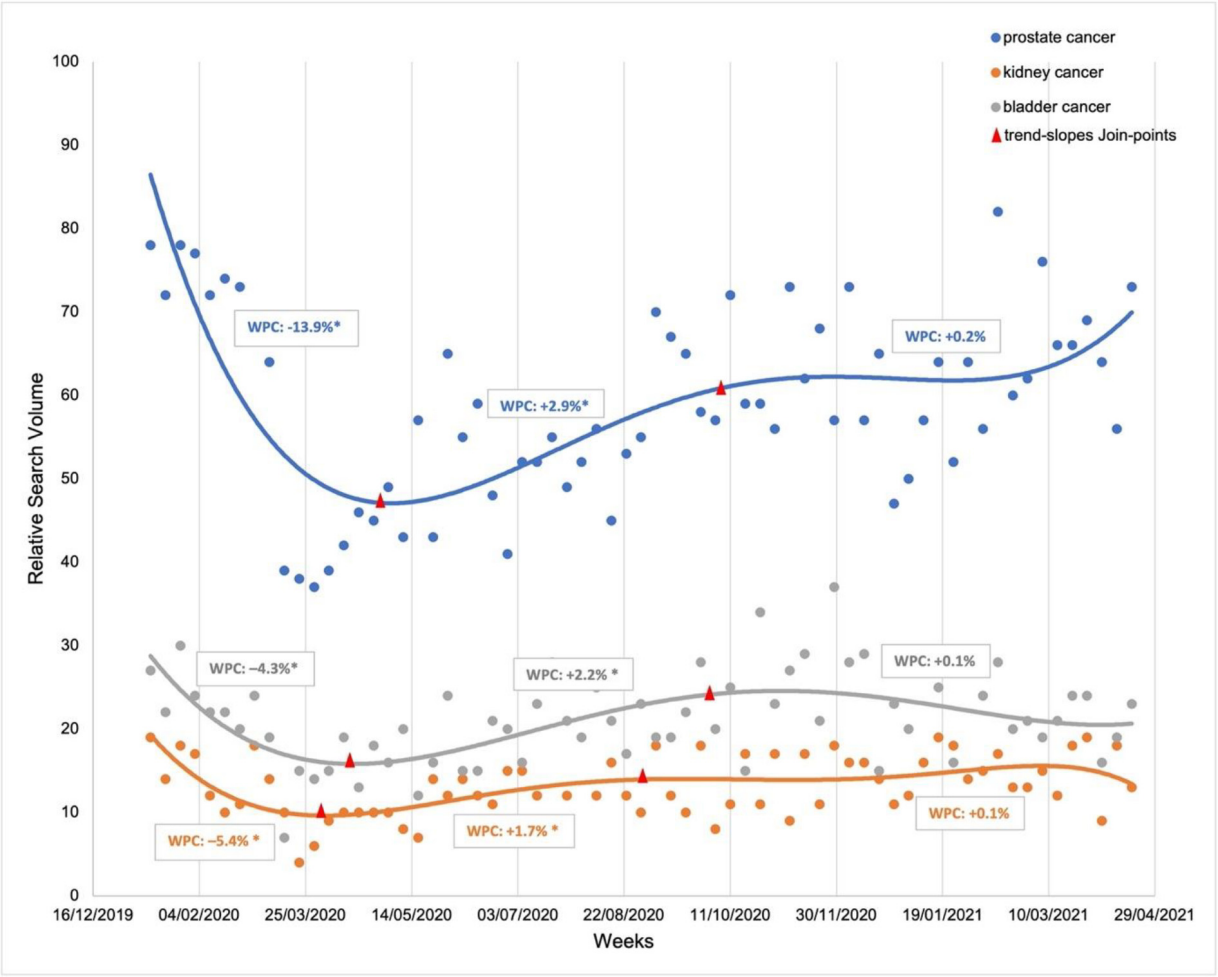
Google Trends relative search volume for urological cancers by week, from January 2020 to April 2021 for the entire world. The asterisk symbol indicates that the week percentage change (WPC) is significantly different from zero at the alpha = 0.05 level.

Worldwide online interest in urological cancers varied significantly during the pandemic. Globally, after the World Health Organization declared COVID-19 pandemic, there was immediate decreased interest for all urological cancers (Fig. 1). The most important decrease was observed for prostate cancer (WPC: –13.9, 95% CI: –22.1; –4.8; *p* = 0.004). The drop for kidney cancer and bladder cancer was less pronounced (WPC: –5.4, 95% CI: –7.6; –3.0; *p* < 0.001, and WPC: –4.3, 95% CI: –6.4; –2.0; *p* < 0.001, respectively). From April 2020, the interest for all three cancers experienced a sharp increase until September 2020 (WPC: +2.9, *p* < 0.001 for prostate cancer; WPC: +1.7, *p* = 0.01 for kidney cancer; and WPC: +2.2, *p* = 0.009 for bladder cancer) followed by a plateau with a statistically nonsignificant increasing trend (all *p* > 0.05). Finally, no interaction between online interest and the season was recorded (all *p* > 0.05).

**Fig. 2:**
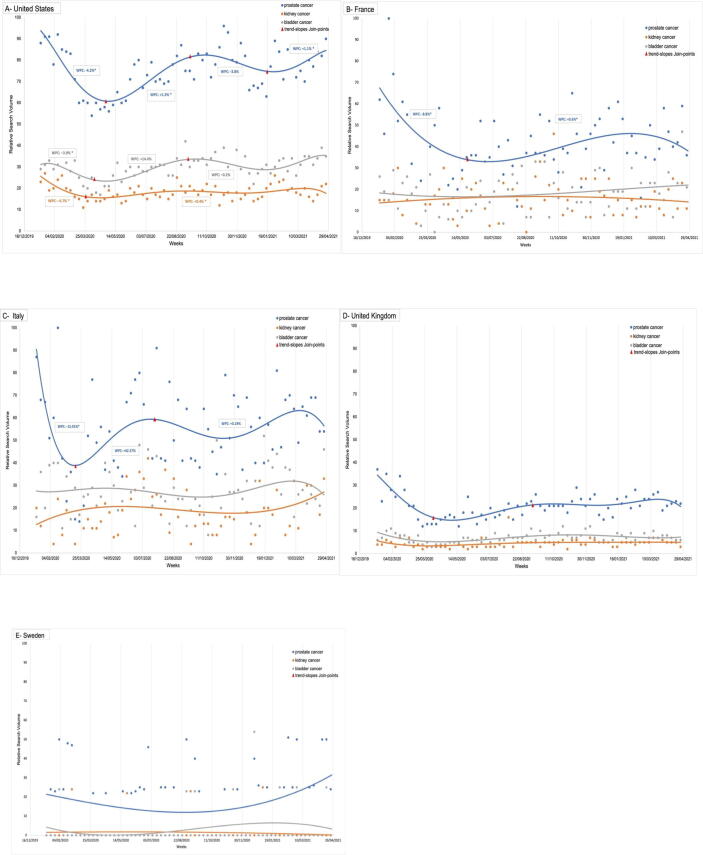
Google Trends relative search volume for genitourinary cancers by week, from January 2020 to April 2021: (A) USA, (B) France, (C) Italy, (D) UK, and (E) Sweden. The asterisk symbol indicates that the week percentage change (WPC) is significantly different from zero at the alpha = 0.05 level.

There were disparities regarding trends across countries. The most important decrease in online search was seen in the USA (Fig. 2A). There was a major drop of interest especially for prostate cancer when the lockdown started in March 2020 (prostate cancer: WPC: –4.2, 95% CI: –5.6; –2.8; *p* < 0.001; kidney cancer: WPC: –4.7, 95% CI: –8; –1.2; *p* = 0.001; bladder cancer: WPC: –3.9, 95% CI: –5.5; –2.4; *p* < 0.001). Then, a significant increase occurred for all terms up to September 2020 followed by a new decreasing trend corresponding to the second wave (autumn 2020) although not significant (all *p* > 0.05).

In France, Italy, and the UK (Fig. 2B–D), the trends varied less sharply. There was no significant variation for kidney and bladder cancer. In France, the interest for prostate cancer diminished during the national lockdown, reaching a lowest rate in May 2020 (WPC: –8.8, 95% CI: –15.1; –2; *p* = 0.01). In Italy, there was a decreasing trend up to April 2020 for prostate cancer (WPC: –15.9, 95% CI: –20.9; –10.6; *p* < 0.01), followed by a spectacular and important revival of interest (WPC: 42.4, 95% CI: –34.7; 210.4; *p* < 0.01). In the UK, there was a decreasing trend from January 2020 to April 2020 for prostate cancer (WPC: –8.6, 95% CI: –11.5; –5.6; *p* < 0.001). After that, a significant increase of interest was observed (WPC: +1.7, 95% CI: 0.7; 2.8; *p* = 0.002) up to September 2020. Finally, in Sweden, no significant variation for prostate, kidney, and bladder cancers was observed.

### 3.2 Comparison with the same period in 2018 and 2019

Table 1 summarises the results of online interest in urological cancers in the whole world, the USA, Italy, the UK, France, and Sweden in the periods before and during COVID-19. In the whole world, the median RSV was significantly lower during the COVID-19 period than in the pre-COVID era for prostate (RSV 60 vs 67.5), kidney (RSV 13 vs 14), and bladder (RSV 19.5 vs 21) cancers.

**Table 1:** Relative search volumes for urological cancers during the COVID-19 pandemic, as compared with the prior 2 yr (2018–2019)

	2018–2019		2020–2021		*p* value
*Whole world*					
Prostate cancer	67.5	(63.5–71)	60	(55.5–63.5)	<0.001
Kidney cancer	14	(13–15)	13	(11–14)	<0.001
Bladder cancer	21	(20–23)	19.5	(17–21)	<0.001
*USA*					
Prostate cancer	81	(77–86)	77	(68.5–83.5)	<0.001
Kidney cancer	21	(19–23)	18	(16–20)	<0.001
Bladder cancer	31.5	(29–34)	30	(27–33)	0.02
*UK*					
Prostate cancer	24	(22–28)	21	(17–24)	0.001
Kidney cancer	5	(4–6)	3	(3–5)	0.001
Bladder cancer	8	(7–9)	7	(5.5–8)	0.001
*Italy*					
Prostate cancer	60	(45–73)	54	(41.5–68)	0.05
Kidney cancer	23	(14–29)	17.5	(12–27)	0.02
Bladder cancer	28	(20–37.5)	26	(21–36)	0.55
*France*					
Prostate cancer	41	(33–52)	39.5	(33.5–52)	0.51
Kidney cancer	27	(26–28)	23.5	(21–26)	0.07
Bladder cancer	18	(17–20)	15	(14–16.5)	0.32
*Sweden*					
Prostate cancer	26	(0–29)	23	(0–25)	0.06
Kidney cancer a	4	(0–53)	2	(0–27)	0.60
Bladder cancer a	5	(0–53)	3	(0–54)	0.17

COVID-19 = coronavirus disease 2019.

a Results are expressed as average and extremes.

We also observed a significant decrease during the COVID-19 pandemic for all urological cancer terms in all four countries. The most significant decrease was seen in the USA and UK (all *p* < 0.05). In Italy, online interest in genitourinary (GU) cancers was also significantly less important during the COVID-19 pandemic: there was a significant decrease of “prostate cancer” and “kidney cancer” search terms (60 vs 54, *p* = 0.05 and 23 vs 17.5, *p* = 0.02, respectively). Bladder cancer interest also diminished but not significantly (28 vs 26; *p* = 0.55). Finally, the interest of the French and Swedish in GU cancers has declined for all terms without being statistically significant (all *p* > 0.05).

### 3.3 Comparison of related searches between the two period (before and during the COVID era)

Regarding search terms associated with urological cancers, there was decreased interest in most assessed categories. The search volume for “prostate cancer prostate-specific antigen” and “prostate cancer treatment” decreased by 3.5% (*p* = 0.10) and 8% (*p* = 0.002), respectively. Paradoxically, we observed a no significant increase about “prostate cancer symptoms” (+1.5%, *p* = 0.25). For the bladder cancer category, the search volume for “bladder cancer symptoms” and “bladder cancer treatment” decreased by 5.5% and 2% (*p* < 0.05), respectively. In contrast, “bladder cancer survival rate” search volume increase by 1.5% (*p* = 0.35). Lastly, a significant decrease in results was observed for the term “kidney cancer symptoms” (–7%; *p* = 0.04) and a no significant decrease in results was observed for “kidney cancer signs” and “kidney cancer treatment” terms (*p* > 0.05; Table 2).

**Table 2:** Relative volumes of most relevant related searches for each urological cancer during the COVID-19 pandemic, as compared with the prior 2 yr (2018–2019)

	2018–2019		2020–2021		*p* value
Prostate cancer symptoms	66	(56–74)	67.5	(58–76)	0.25
Prostate cancer PSA	29	(24–35)	25.5	(19.5–34.5)	0.1
Prostate cancer treatment	62.5	(51–70.5)	54.5	(47–64.5)	0.002
Bladder cancer symptoms	57.5	(47.5–64.5)	52	(42.5–62.5)	0.04
Bladder cancer treatment	21	(16.5–25)	19	(13–25)	0.003
Bladder cancer survival rate	3	(2–6)	4.5	(2–8)	0.35
Kidney cancer symptoms	67	(51.5–72.5)	60	(44–70.5)	0.04
Kidney cancer signs	21	(15–30)	18.5	(13–27)	0.33
Kidney cancer treatment	14	(8–20)	12	(8–17)	0.12

COVID-19 = coronavirus disease 2019; PSA = prostate-specific antigen.

## 4 Discussion

In this study, we used a publicly available big data tool to explore the interest in search terms related to urological cancers during the COVID-19 pandemic. Our results suggest that awareness of urological cancers was significantly reduced worldwide, especially in the USA.

Over the past decades, the Internet has transformed our access to information. Google is undoubtedly the most famous search engine and represents a resource for patients to better understand their disease, find support, and make decisions. In the field of oncology, it has been used to evaluate the impact of cancer awareness campaign [9], [10] and public interest in different oncology treatments [11]. In addition, some teams showed an association between Google search activity, cancer incidence [12], and mortality rates [13] of some malignancies.

Our analysis shows a reduction of online interest for urological cancers during the COVID-19 pandemic. These results have to be compared with a simultaneous decline of cancer diagnoses [14]. Almost 90% of prostate cancers are detected by screening in the USA [15]. During the COVID-19 pandemic, prostate-specific antigen screening decreased by 60%, which for example resulted in a 30% decrease in diagnosis in the state of Massachusetts [16]. This lower incidence might explain the diminution in Internet search. Moreover, radiological imaging declined after the American College of Radiology encouraged the rescheduling of nonurgent and non–COVID-related outpatient visits [17]. Since renal cell carcinomas are incidentally discovered in the majority of cases in industrialised countries, it was highly expected that the incidence of renal cell carcinoma would decrease [18].

There was also a significant drop of interest in bladder cancer mostly in the USA. This is a matter of concern since it has been established that the risk of death from bladder cancer increases with the delay between symptoms and diagnosis [19]. Moreover, bladder cancer occurs mostly in elderly patients [20] who have particularly been reluctant to consult their doctors due to the fear of COVID-19. As a result, we could observe a rise in bladder cancer deaths in the coming months.

The projected incidence and mortality rates due to cancers in the post-COVID era will likely rise. In the UK, it has been estimated that there could be between 361 and 3621 additional deaths linked to cancer because of diagnosis delays during lockdown [21], [22]. In addition, lockdown restrictions and closure of nonessential businesses reduced salaries and increased the unemployment rate [23]. It has been shown that for every 1% increase in unemployment, there was a 2% decrease of cancer incidence and surgical procedures [24]. It is estimated that the 2008 economic crisis has caused an additional 260 000 cancer-related deaths [25]. Cancer diagnoses could grow in the coming months, and future studies will show us the impact of diagnosis delays on oncological outcomes.

We do not know exactly what impact COVID-19 will have on the epidemiology of urological cancers. We found a significant lack of interest in public for the three major GU cancers. One reason for the disinterest of public is that individuals were spending time exploring COVID-19 rather than cancer symptom information when the pandemic began due to it being a new and previously unheard-of topic that caught public attention. Alternatively, the decrease in interest may have been related to the lack of ability to plan screening and care services even for patients who were motivated to get it, even though the rapid implementation of telehealth and triage reprioritisation of care within uro-oncological department have been able to offer permanent value in enhancing cancer care quality and access [26]. Telemedicine and teleconferencing can partly substitute the patient-physician relation for the short term [27]. However, it cannot replace major steps in the diagnostic and treatment processes of cancers (eg, haematuria requiring cystoscopy for a diagnosis of bladder cancer). In addition, this technology has some limitations including acceptance by patients and doctors, financial burden, medicolegal concerns, and continuous training for effective usage [28]. As an end of the preventive measures against COVID-19 remains unlikely in the short-term future, telehealth can contribute to providing needed care for cancer patients even if the long-term efficacy and safety are unknown. To keep up to date with the latest information, we provide an online tool that can easily be accessed for readers to check trends in real time: https://urologie-rennes.fr/trends.

Some limitations of our study must be underlined. First, we do not have any knowledge regarding the individuals performing searches: the internaut can be the patient himself, the family, or a health worker. Consequently, it is hazardous to draw any formal causal relationships even though the amount of data can show us an epidemiological trend. Second, worldwide evaluation was limited by disparities across countries regarding Internet access, and English speakers were inevitably over-represented. To account for this, at least in part, we used search terms that appear as “topics” on Google Trends, which means that the search includes the same or very similar terms in other languages, as well as variations of the search term. Third, given Google’s algorithm for normalising the search volume frequency, the absolute search volume for specific terms is unavailable. However, some authors found that the RSV provided by Google corresponded well with the degree of online awareness [29]. Fourth, because of the very low incidence, some urological cancers, such as testicular and penile cancers, were not analysed. The search terms were selected to be as popular as possible. We cannot confirm that all search activity for the selected terms exclusively reflects health search behaviour. Finally, our study was restricted to Google users and does not consider individuals who use other search engines, notably those available in China.

## 5 Conclusions

In conclusion, we found that online public interest in urological cancers decreased during the COVID-19 pandemic. There is some uncertainty regarding the consequences of this unique health care scenario, and future data will show us the impact of diagnosis and treatment delays on oncological outcomes.

  ***Author contributions*:** Zine‐Eddine Khene had full access to all the data in the study and takes responsibility for the integrity of the data and the accuracy of the data analysis.

*Study concept and design*: Z.-E. Khene, Guérin, F. Khene, Pradère, Roumiguié, Mathieu, , Borchiellini, Pignot, Neuzillet, Ploussard, Bigot, Rouprêt, Bensalah.

*Acquisition of data*: Z.-E. Khene, Guérin, F. Khene.

*Analysis and interpretation of data*: Z.-E. Khene, Guérin, F. Khene, Bensalah.

*Drafting of the manuscript*: Z.-E. Khene, Guérin, F. Khene.

*Critical revision of the manuscript for important intellectual content*: Z.-E. Khene, Guérin, F. Khene, Pradère, Roumiguié, Mathieu, , Borchiellini, Pignot, Neuzillet, Ploussard, Bigot, Rouprêt, Bensalah.

*Statistical analysis*: Z.-E. Khene, F. Khene.

*Obtaining funding*: None.

*Administrative, technical, or material support*: None.

*Supervision*: Roumiguié, Mathieu, , Borchiellini, Pignot, Neuzillet, Ploussard, Bigot, Rouprêt, Bensalah.

*Other*: None.

  ***Financial disclosures:*** Zine‐Eddine Khene certifies that all conflicts of interest, including specific financial interests and relationships and affiliations relevant to the subject matter or materials discussed in the manuscript (eg, employment/affiliation, grants or funding, consultancies, honoraria, stock ownership or options, expert testimony, royalties, or patents filed, received, or pending), are the following: None.

  ***Funding/Support and role of the sponsor*:** None.
